# Propofol Exposure in Early Life Induced Developmental Impairments in the Mouse Cerebellum

**DOI:** 10.3389/fncel.2017.00373

**Published:** 2017-11-22

**Authors:** Rui Xiao, Dan Yu, Xin Li, Jing Huang, Sheng Jing, Xiaohang Bao, Tiande Yang, Xiaotang Fan

**Affiliations:** ^1^Department of Anesthesiology, Xinqiao Hospital, Third Military Medical University, Chongqing, China; ^2^Department of Developmental Neuropsychology, Third Military Medical University, Chongqing, China

**Keywords:** propofol, cerebellum, development, neurotoxicity, mouse

## Abstract

Propofol is a widely used anesthetic in the clinic while several studies have demonstrated that propofol exposure may cause neurotoxicity in the developing brain. However, the effects of early propofol exposure on cerebellar development are not well understood. Propofol (30 or 60 mg/kg) was administered to mice on postnatal day (P)7; Purkinje cell dendritogenesis and Bergmann glial cell development were evaluated on P8, and granule neuron migration was analyzed on P10. The results indicated that exposure to propofol on P7 resulted in a significant reduction in calbindin-labeled Purkinje cells and their dendrite length. Furthermore, propofol induced impairments in Bergmann glia development, which might be involved in the delay of granule neuron migration from the external granular layer (EGL) to the internal granular layer (IGL) during P8 to P10 at the 60 mg/kg dosage, but not at the 30 mg/kg dosage. Several reports have suggested that the Notch signaling pathway plays instructive roles in the morphogenesis of Bergmann glia. Here, it was revealed that propofol treatment decreased Jagged1 and Notch1 protein levels in the cerebellum on P8. Taken together, exposure to propofol during the neonatal period impairs Bergmann glia development and may therefore lead to cerebellum development defects. Our results may aid in the understanding of the neurotoxic effects of propofol when administrated to infants.

## Introduction

The cerebellum is characterized by laminated structures, and its abnormal morphogenesis may lead to deficits related to disorders such as Dandy-Walker Malformations, Joubert Syndrome and other congenital spinocerebellar ataxias (Millen and Gleeson, 2008). An increasing number of recent studies have suggested that anesthesia may be neurotoxic to the brain and lead to various long-term behavioral disorders, especially in the infants (Olney et al., 2002; Stargatt et al., 2006; Patel and Sun, 2009; DiMaggio et al., 2011; Reddy, 2012; Sinner et al., 2014).

Proper cerebellar function depends on well-organized neuronal connections and the integration of afferent and efferent fibers into the cerebellar circuitry. The mouse cerebellar cortex has a well-defined architecture consisting of the following three major layers: (1) the molecular layer (ML); (2) the Purkinje cell layer (PCL); and (3) the granule layer (Voogd and Glickstein, 1998). To establish proper lamination and circuitry, important events such as neuronal differentiation, morphogenesis, and migration need to be precisely regulated during cerebellar development (Altman and Winfree, 1977; Buffo and Rossi, 2013). Mouse cerebellar development continues until 3 weeks after birth. During this postnatal period, cerebellar cells undergo sequential development steps in spatially well-defined regions. Purkinje cells are the principal neurons, and are transformed from a stellate morphology into their essential dendritic structures between the first and second weeks (Eccles, 1970; Sotelo and Rossi, 2013). At the early postnatal stage, granule cell precursors are the most abundant in the external granular layer (EGL), followed by an inward radial migration along the Bergmann glial radial fibers to their destination, the internal granule layer (IGL; Komuro et al., 2001; Buffo and Rossi, 2013). By the end of the third postnatal week, the EGL disappeared and three well-defined neuronal layers have formed (Qiu et al., 2010).

Propofol (6,2 diisopropylphenol) is an anesthetic that works through the activation of gamma amino butyric acid A (GABA_A_) receptors and block of N-methyl-D-aspartate (NMDA) glutamate receptors (Franks and Lieb, 1994; Irifune et al., 2003). It is widely used in the clinic for induction and maintenance of general anesthesia and conscious sedation, especially in neurosurgery, for its unique benefit on cerebral physiology including reduction in cerebral blood flow, intracranial pressure and cerebral metabolism (Diaz and Kaye, 2017). The increasing utilization of propofol as a drug of abuse is of high concern, and a public health threat, especially for developing fetuses. Studies in rodents have confirmed that propofol exposure caused toxic effects in the developing brain. Consistent with other reports, we previously found that propofol administration during early postnatal life suppressed hippocampal neurogenesis (Huang et al., 2016). Additionally, propofol has been implicated in causing movement disorders since 30 years ago, which strongly suggests that propofol may damage the cerebellum (Dingwall, 1987; Zabani and Vaghadia, 1996; Bendiksen and Larsen, 1998; Brooks, 2008). Recent studies have also indicated that propofol depressed Purkinje cell activity and affected the cerebellum circuitry (Jin R. et al., 2015; Jin W. Z. et al., 2015; Lee K. Y. et al., 2015). However, little is known regarding the impact of propofol exposure on the development of the cerebellar neuronal population.

In this study, newborn mice at P7 were administered a single injection of 30 or 60 mg/kg of propofol or vehicle of equal volume to explore the effects of propofol on Purkinje cell dendritogenesis and Bergmann glia development. The migration of newborn granule cells in the EGL was evaluated with a 5-bromodeoxyuridine (BrdU) labeling protocol. As the Notch signaling pathway has been indicated to play crucial roles in the regulation of development of Bergmann glia, its involvement in the underlying mechanism on the neurotoxic effects by propofol during the early stage were also explored.

## Materials and Methods

### Animals

Male and female C57/BL6 mice were provided by the Third Military Medical University and housed under a 12 h light/dark cycle in a temperature-controlled room with free access to food and water. All the experimental procedures were performed in accordance with the guidelines for laboratory animal care and use and were approved by Third Military Medical University. Each litter was kept together with its mother throughout the experiment, except for the brief intervals of separation required for the daily injections. At least five mice in each group were analyzed for immunofluorescence staining and three mice for western blot.

### Drug Treatment

The day of birth was designated postnatal day 0 (P0). On P7, pups received a vehicle or propofol injection intraperitoneally (i.p.) at a subanesthetic dose of 30 or 60 mg/kg (Cattano et al., 2008; Yang B. et al., 2014), according to our previous study (Huang et al., 2016). The same volume of intralipid was administered i.p. as a vehicle control for propofol. All the neonatal mice were grouped randomly with a random number table base for similar body weight.

To explore the morphological changes in the Purkinje cells, Bergmann glia and granule neuron, pups were sacrificed 24 h (P8) after drug treatment. To evaluate whether propofol affected the radial migration of the granule neurons, a single-dose BrdU injection (50 mg/kg i.p., dissolved in saline) was administered to the pups at P8, which was 1 day after injection with propofol or vehicle. Pups were sacrificed 2 days after the BrdU injection (P10).

To maintain a mouse body temperature of 37°C, the pups were primitively anesthetized in their home cage and then transferred to a Thermocare^®^ ICS therapy warmer unit (Thermocare, Incline Village, NV, USA) after being sedated to keep warm in all the experiments. Meanwhile, mouse normal skin color and respiration were observed.

### Immunofluorescence

The dissected cerebella were soaked in 4% paraformaldehyde for 24 h. For cryosections, the tissues (P8) were embedded and sectioned in the sagittal plane at 30 μm. The remaining tissues (P8) and tissues collected on P10 were embedded in paraffin and sagittal sections (5 μm thickness) were collected. Cryosections were used for all the immunofluorescence staining for P8 and paraffin sections were used for Hematoxylin-eosin (HE) staining and BrdU immunofluorescence staining. The sections were pretreated with 3% bovine serum albumin (BSA) (37°C, 1 h) to block non-specific binding and 0.3% Triton X-100 (37°C, 30 min) to increase permeability. Then, the sections were incubated with the following primary antibodies in 1% BSA (4°C, 18 h): (1) mouse anti-calbindin D-28K (CB) (1:1000, Swant, Bellinzona, Switzerland); (2) rabbit polyclonal anti-glial fibrillary acidic protein (GFAP) (1:200, Merck Millipore, Darmstadt, Germany); (3) rabbit polyclonal anti-brain lipid binding protein (BLBP) (1:400, Merck Millipore, Darmstadt, Germany); and (4) mouse anti-neuronal nuclei (NeuN) (1:200, Merck Millipore, Darmstadt, Germany). One percent BSA served as the negative control. After three washing steps with phosphate-buffered saline (PBS, pH 7.4), the sections were incubated with the following secondary antibodies in PBS (room temperature (RT), 3 h): (1) Alexa Fluor 488-conjugated anti-mouse IgG (1:400, Jackson ImmunoResearch, West Grove, PA, USA) for CB and NeuN staining; and (2) cy3-conjugated anti-rabbit IgG (1:400, Jackson ImmunoResearch, West Grove, PA, USA) for BLBP and GFAP staining. All the sections were counterstained with 4′,6-diamidino-2-phenylindole (DAPI) (Sigma-Aldrich, St. Louis, MO, USA) and then mounted in Vectashield (Vector Laboratories, Burlingame, CA, USA). For BrdU staining, the paraffin sections were deparaffinized in xylene, rehydrated in graded alcohol and processed for antigen retrieval by boiling in citrate buffer (pH 6.0) for 5 min. After incubation in 2 M HCl (37°C, 30 min) and 0.3% Triton X-100 (37°C, 30 min), the sections were exposed to mouse anti-BrdU IgG (37°C for 2 h and then RT for 22 h) (1:600, BD Pharmingen™, Palo Alto, CA, USA) in 1% BSA, followed by the cy3-conjugated anti-mouse IgG secondary antibody (RT, 3 h) (1:400, Jackson ImmunoResearch, West Grove, PA, USA) and DAPI counterstaining. Fluorescence micrographs of the whole parasagittal cerebellar slices were acquired under a Zeiss (Oberkochen, Germany) Axiovert microscope equipped with a Zeiss AxioCam digital color camera connected to the Zeiss Axiovision 3.0 system. The pictures of the Purkinje dendrite and Bergmann fiber contact points were taken with a TCS-SP8 (Leica, Germany) laser scanning confocal microscope connected to a LAS AF Lite system. A z-stack of images, consisting of 6 image planes taken at 1 μm interval was obtained (for a total stack depth of 5 μm). The 5 μm z-stack was taken from the middle of the section to minimize the potential artificial bias.

### Western Blot

Cerebella were harvested on P8 and then isolated and homogenized in ice-cold RIPA Lysis buffer (Beyotime, Shanghai, China). After centrifuging the lysates (15,000× *g*, 5 min at 4°C), the protein concentration was calculated using the Bicinchoninic Acid Kit (Beyotime, Shanghai, China). Then, 50 μg of protein from each sample was separated by 10% SDS-polyacrylamide electrophoresis (120 min 80 V) and then transferred to a nitrocellulose (NC) membrane (90 min at 210 mA). The membranes were incubated in 5% fat-free milk in Tris-buffered saline containing 0.1% Tween 20 (3 h at RT). Membranes were then incubated with the following primary antibodies (4°C, overnight): (1) hamster monoclonal anti-Notch1 (1:500, Santa Cruz Biotechnology, Santa Cruz, CA, USA); (2) rabbit polyclonal anti-Jagged1 (1:500, Santa Cruz Biotechnology, USA); (3) mouse anti-β-actin (1:1000, Cell Cwbio, Beijing, China); and (4) rabbit anti-GAPDH (1:1000, Cell Cwbio, China), followed by the following peroxidase-conjugated secondary antibodies (RT, 2 h): (1) goat anti-mouse IgG (1:1000, Santa Cruz Biotechnology, USA); (2) goat anti-rabbit IgG (1:1000, Santa Cruz Biotechnology, USA); and (3) goat anti-Syrian hamster IgG (1:1000, Santa Cruz Biotechnology, USA). All the bands were exposed to X-ray films (Kodak, Rochester, NY, USA), detected using an enhanced chemiluminescence detection kit (Pierce, Rockford, IL, USA), and analyzed with the Gel-Pro analyzer (Quantity One 4.0; Bio-Rad Laboratories, Hercules, CA, USA). Quantification of Jagged1 and Notch1 were normalized to the internal reference protein β-actin or GAPDH, and then normalized to the control values.

### Quantification

The quantification was obtained from regional analysis of lobe IX. All the sections were taken from similar medial-lateral position within the cerebellum, and each count area was chosen from the same field by the middle of the lobe IX. Calbindin-positive cells were analyzed along the long axis for 500 μm in the middle, and the dendrite length of Purkinje cells was evaluated by measuring the primary dendrite from the soma up to the surface of the ML (three Purkinje dendrites were measured per picture). Number of the NeuN-positive granule neurons were analyzed in the center region of the IGL in lobe IX along the long axis (unit area 2000 μm^2^). The number of BLBP- and GFAP-positive Bergmann fibers was counted from a 100-μm length in the middle area of lobe IX according to our previous methods (Yamada et al., 2000; Eiraku et al., 2005; Yang Y. et al., 2014). To analyze the astrocytes in the deep white matter, we compared the intensity of the GFAP-positive cells and fibers. Both the background integrated optical density (IOD) and surveyed area (same center area of the white matter from each group) were acquired, and the relative optical density (ROD) was calculated by subtracting the background from the IOD of the positive staining (Bao et al., 2017). Contact points between the calbindin-positive Purkinje cells and GFAP-positive Bergmann fibers were defined as where the tips of growing Purkinje cell dendrites were aligned parallel and attached directly to the rod-like domain of Bergmann fibers, entering the base of the overlying EGL, as previously reported (Yamada et al., 2000; Yamada and Watanabe, 2002; Lordkipanidze and Dunaevsky, 2005). Points were counted per image (212.5-μm length) at the interface between the EGL and ML. Only the yellow dots at the end of the dendrites in the direction of the Bergmann fiber were included, while the crossed ones were excluded in case of false positive. For quantifying granule neuron migration, BrdU-labeled cells were counted in a rectangular box (200 μm width and about 100 cells were counted) extending from the pial surface to the end of the IGL; this value was expressed as a percentage of the total number of BrdU-labeled cells. At least five sections were analyzed in each mouse and five mice from each group. All the quantitative statistics were performed blind to the experimental treatment.

### Statistical Analysis

All the data were presented as the mean ± standard deviation and analyzed using one-way analysis of variance (ANOVA) followed by Fisher’s protected least- significant difference *post hoc* test or a least-significant difference multiple-comparison. The differences were statistically significant when the *P* value was less than 0.05. Statistical analysis was performed using the SPSS 19.0 software (SPSS Inc., Chicago, IL, USA).

## Results

### Propofol Treatment Does Not Alter Cerebellum Formation

**Figure 1:**
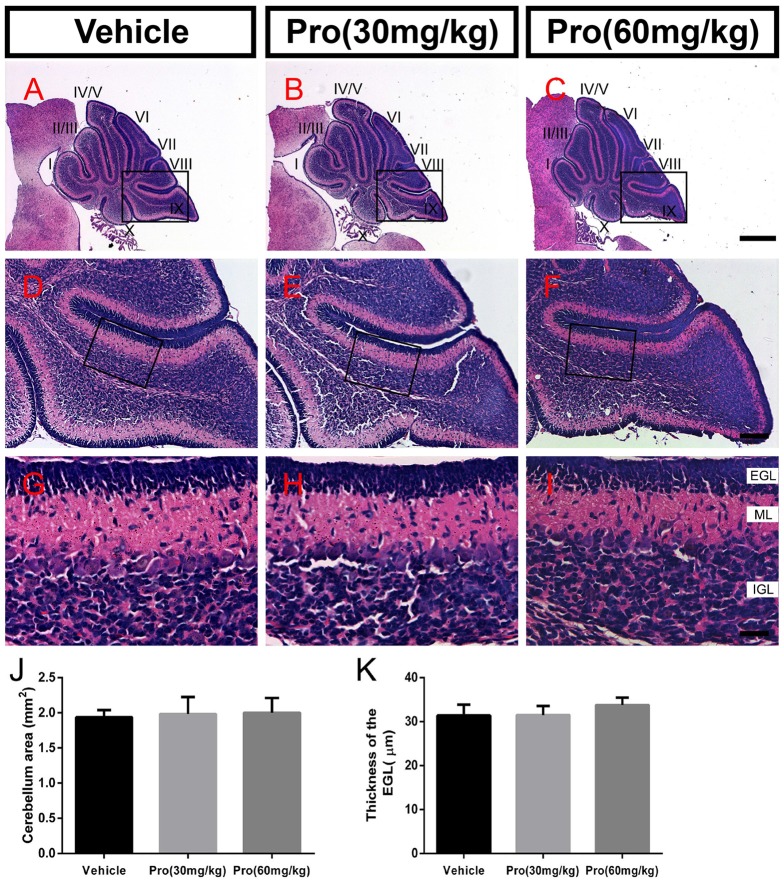
Propofol treatment did not alter the formation of the cerebellum at P8. **(A–C)** The folia structure of the cerebellum at P8 is revealed by Hematoxylin-eosin (HE) staining. **(D–F)** Magnifications of panels **(A–C)** show the structure of lobe IX. **(G–I)** Magnified of the area identified by the black boxes in panels **(E–H)** show the external granular layer (EGL), molecular layer (ML) and internal granule layer (IGL) of lobe lobe IX. **(J)** Propofol treatment did not alter the morphologies or cerebellar areas at P8 between the groups. **(K)** Comparison of relatively identical areas from lobe IX show no obvious differences in the thickness of the EGL at P8 between the groups. Data are presented as the mean ± SD (*n* = 4). Scale bar: **(A–C)**: 500 μm; **(D–F)**: 100 μm; and** (G–I)**: 25 μm.

Folia structure revealed by the HE staining on the sagittal vermal sections from the cerebellum was similar in all groups at P8 (Figures 1A–I). There were no significant alterations in the areas (Vehicle 1.94 ± 0.10 mm^2^, Pro 30 mg/kg or 60 mg/kg 1.98 ± 0.24 mm^2^ or 2.00 ± 0.21 mm^2^, respectively, *P* > 0.05; *n* = 4; Figure 1J). In the same area of lobe IX (Figures 1D–F), EGL thickness was not altered by propofol treatment (Vehicle 31.39 ± 2.46 μm; Pro 30 mg/kg or 60 mg/kg 31.48 ± 2.08 μm or 33.75 ± 1.69 μm, respectively, *P* > 0.05; *n* = 4; Figure 1K).

### Propofol Treatment Decreases the Number of and Dendrite Outgrowth from Purkinje Cells in a Dose-Dependent Manner

**Figure 2:**
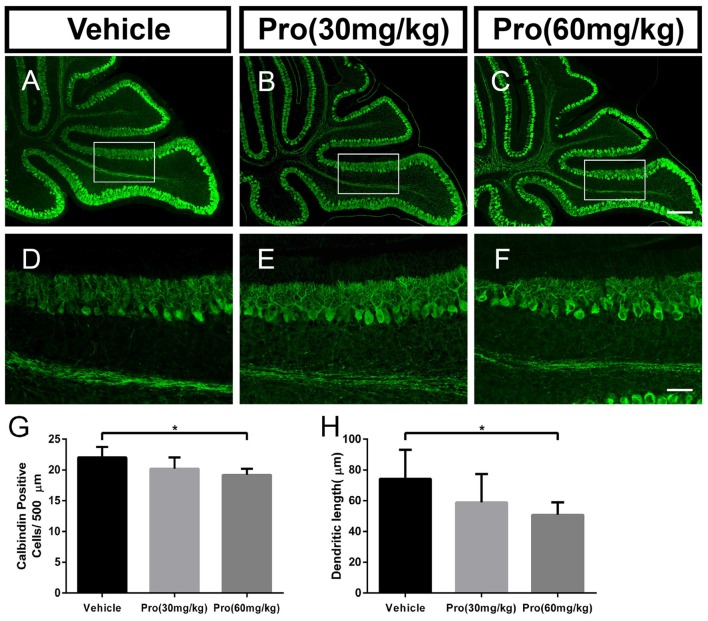
Propofol treatment decreased the number of Purkinje cells and depressed the dendrite length at P8. **(A–C)** Calbindin-stained cerebellar Purkinje cells from the **(A)** Vehicle, **(B)** Propofol (30 mg/kg) and **(C)** Propofol (60 mg/kg) groups. **(D–F)** Magnified images of Panels **(A–C)** show the calbindin-positive cells and their dendrites in lobe IX. **(G)** Quantification of the number of calbindin-positive cells in the purkinje cell layer (PCL). **(H)** Quantification of the primary Purkinje dendrite length. Data are presented as the mean ± SD (*n* = 5). Scale bar: **(A–C)**: 200 μm and **(D–F)**: 50 μm. *P < 0.05.

Purkinje cells were revealed by their specific marker calbindin. In comparably identical middle areas from lobe IX in the cerebellum (Figures 2A–F), there was no significant difference in the number of calbindin-positive cells (Figure 2G) and the Purkinje dendrite length (Figure 2H) between the pups treated with 30 mg/kg propofol and pups treated with vehicle. However, pups treated with 60 mg/kg propofol had decreased calbindin-positive cells compared to the pups treated with vehicle (Vehicle 22.04 ± 1.69 and Pro 60 mg/kg 19.20 ± 1.00, *P* < 0.05; *n* = 5; Figure 2G). The Purkinje dendrite length was also shortened by 60 mg/kg propofol treatment (Vehicle 74.33 ± 18.77 μm and Pro 60 mg/kg 50.85 ± 8.12 μm, *P* < 0.05; *n* = 5; Figure 2H).

### Propofol Treatment Does Not Alter NeuN-Positive Granule Neurons in the IGL

**Figure 3:**
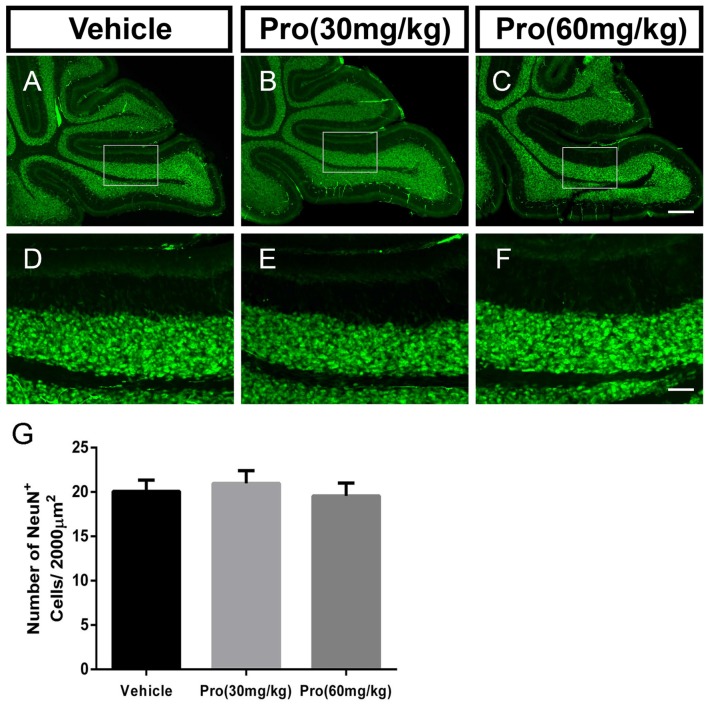
Propofol treatment did not affect the NeuN-positive cells in the IGL at P8. **(A–C)** NeuN-stained cerebellar granule neurons from the **(A)** Vehicle, **(B)** Propofol (30 mg/kg) and **(C)** Propofol (60 mg/kg) groups. **(D–F)** Magnified images of panels **(A–C)** show the NeuN-positive cells in lobe IX. **(G)** Quantification of the NeuN positive cells in the IGL. Data are presented as the mean ± SD (*n* = 5). Scale bar: **(A–C)**: 200 μm and **(D–F)**: 50 μm.

NeuN is a specific marker for granule neurons located in the IGL of the cerebellum. In comparably center areas of the IGL from lobe IX in the cerebellum (Figures 3A–F), propofol treatment at both doses of 30 mg/kg and 60 mg/kg did not alter the number of NeuN-positive cells in the IGL when compared to vehicle treatment (Vehicle 20.08 ± 1.25, Pro 30 mg/kg or 60 mg/kg 20.96 ± 1.45 or 19.56 ± 1.44, respectively, *P* > 0.05; *n* = 5; Figure 3G).

### Propofol Treatment Suppresses Bergmann Glial Cell Filiform Processes and Affects the Astrocyte Phenotype

**Figure 4:**
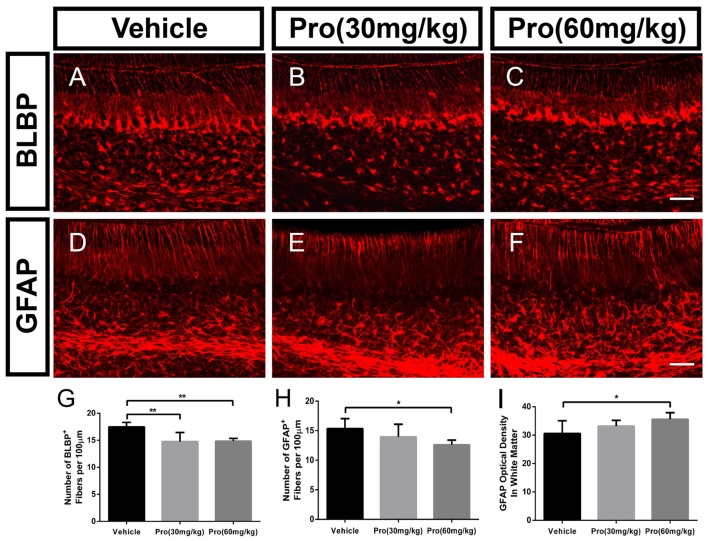
Propofol treatment suppressed Bergmann glial cell filiform processes at P8. **(A–C)** Brain lipid binding protein (BLBP)-stained Bergmann glia fibers in lobe IX from the **(A)** Vehicle, **(B)** Propofol (30 mg/kg) and **(C)** Propofol (60 mg/kg) groups. **(D–F)** Glial fibrillary acidic protein (GFAP)-stained Bergmann glia fibers in lobe IX from the **(D)** Vehicle, **(E)** Propofol (30 mg/kg) and **(F)** Propofol (60 mg/kg) groups. **(G)** Quantification of the number of BLBP-positive fibers in the ML. **(H)** Quantification of the number of GFAP-positive fibers in the ML. **(I)** Quantification of the optical density of GFAP-positive staining in the white matter. Data are presented as the mean ± SD (*n* = 5). Scale bar: **(A–F)**: 50 μm. *P < 0.05 and **P < 0.01.

Bergmann glia was a specialized form of astrocyte, derived from radial glial cells (Xu et al., 2013). During the first week of postnatal development, Bergmann glia were located among Purkinje cells and extended fibers into the ML directing the distal growth of the Purkinje cell dendritic tree (Voogd and Glickstein, 1998; Yamada and Watanabe, 2002). To determine whether propofol treatment affected Bergmann glial shafts, we observed Bergmann glial organizational structure in lobe IX of the cerebellum through immunostaining assays with specific antibodies for BLBP and GFAP. The radial processes from the BLBP-positive Bergmann fibers were found to extend to the surface of the cerebellum, and BLBP-positive Bergmann soma aligned roughly in a single layer next to Purkinje cells (Figures 4A–C). The BLBP-stained radial fibers were decreased following propofol treatment (30 or 60 mg/kg) compared with the vehicle-treated group (Figure 4G). Bergmann glial morphology was further assessed by immunostaining histological sections of the cerebella with antibodies directed at GFAP (Figures 4D–F). There were no significant differences in the GFAP-labeled Bergmann glial processes between the propofol (30 mg/kg)-treated and vehicle-treated groups, whereas the number of radial shafts was significantly decreased after propofol administration at the 60 mg/kg dose; and the images displayed hyperplastic astrocytes in the deep white matter (Figures 4H,I). The results showed that propofol promoted a great disturbance in radial glia phenotypic differentiation and Bergmann glia filiform processes that influenced the formation of the radial scaffold.

### Propofol Treatment Disrupts the Contacts between Purkinje Cells and Bergmann Glial Cells

**Figure 5:**
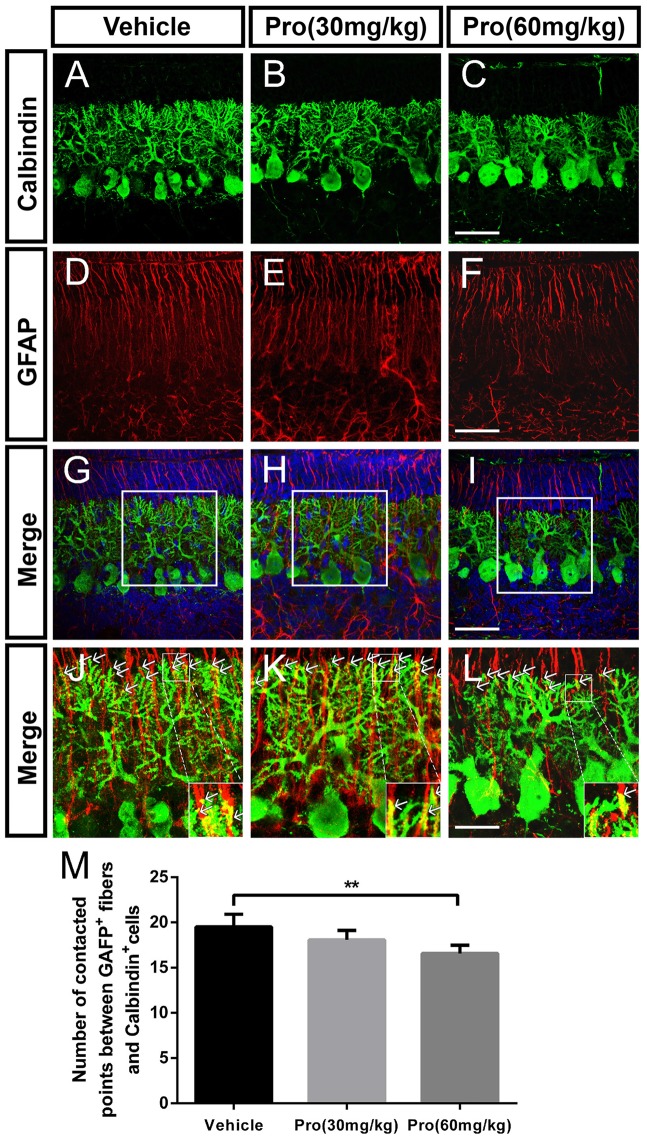
Propofol treatment disrupted the contacts between Purkinje cells and Bergmann glial cells at P8. **(A–L)** Immunolabeling for calbindin (green), GFAP (red), 4′,6-diamidino-2-phenylindole (DAPI) (blue), their merged images and respective high-resolution images of the merges in the cerebellar lobe IX. **(A–C)** Calbindin-stained Purkinje cells from the **(A)** Vehicle, **(B)** Propofol (30 mg/kg) and **(C)** Propofol (60 mg/kg) groups. **(D–F)** GFAP-stained Bergmann glial cell fibers from the **(D)** Vehicle, **(E)** Propofol (30 mg/kg) and **(F)** Propofol (60 mg/kg) groups. **(G–I)** The merged images showing the calbindin staining, GFAP staining and DAPI in the PCL. **(J–L)** Magnified images of panels **(G–I)** show the relationship between the calbindin-positive cells and GFAP-positive cells. The arrows indicate that the tips of calbindin-immunopositive dendrites are intimately attached to the rod-like shaft of Bergmann fiber contacting domains **(M)** Quantification of the numbers of contact points between the GFAP-positive fibers and calbindin-positive cells around the border between the ML and EGL in the identical lobe of the cerebellum. Data are presented as the mean ± SD (*n* = 5). Scale bar: **(A–I)**: 50 μm and **(J–L)**: 25 μm. **P < 0.01.

Purkinje cells make contacts with Bergmann glia through the connections between Purkinje cell dendrites and Bergmann glia fibers in the ML (Figures 5A–I). Connections were defined as dendritic tips in parallel and closely stuck to the Bergmann glia fibers at the interface between the ML and EGL (Figures 5J–L). Immunolabeling for calbindin-positive dendrites and GFAP-positive fibers revealed that the number of contact points was significantly decreased due to the high propofol treatment at 60 mg/kg compared with the vehicle-treated group (Vehicle 19.50 ± 1.40 and Pro 60 mg/kg 16.58 ± 0.90, *P* < 0.01; *n* = 5; Figures 5J–M). The number of contact points was not affected after administration of 30 mg/kg propofol to the mice. Arrows showed the tips of calbindin-immunopositive dendrites are intimately attached to the rod-like shaft of Bergmann fiber contacting domains. These data indicated that the attachments of the Purkinje cells on Bergmann fibers were disrupted after propofol injection, which subsequently resulted in Purkinje cell dendritogenesis disorder.

### Propofol Treatment Delays the Migration of Granule Neurons from the EGL to IGL

**Figure 6:**
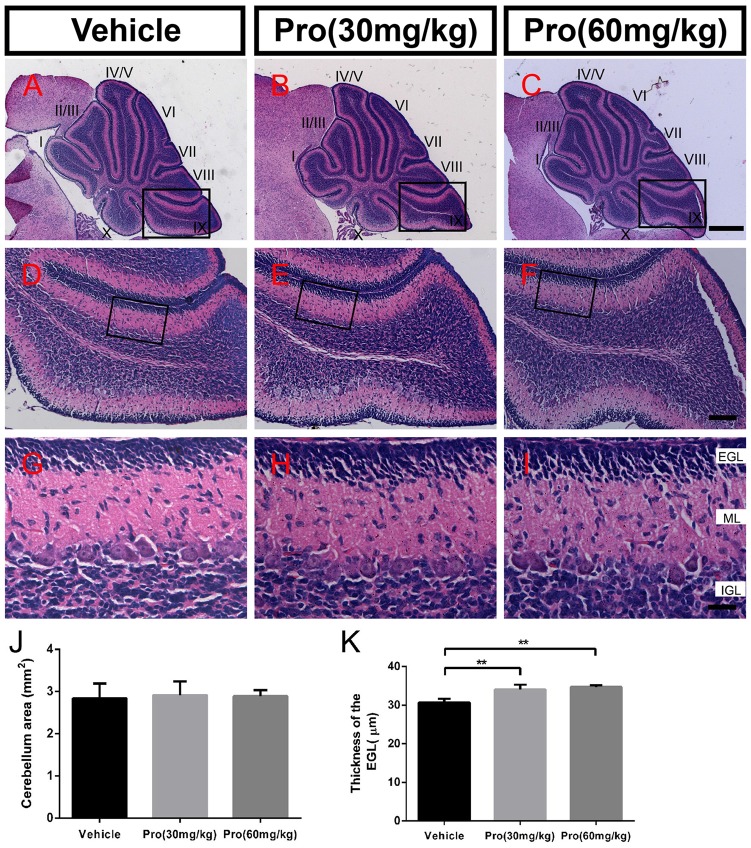
Propofol treatment increased the thickness of the EGL at P10. **(A–C)** The folia structure of the cerebellum is revealed by HE staining from the **(A)** Vehicle, **(B)** Propofol (30 mg/kg) and **(C)** Propofol (60 mg/kg) groups. **(D–F)** Magnified images of panels **(A–C)** show the structure of lobe IX. **(G–I)** Magnified areas identified by the black boxes in panels **(D–F)** show the EGL, ML and IGL, respectively, from lobe IX. **(J)** Propofol treatment did not alter the morphology or cerebellar area at P10 between the groups.** (K)** Comparison of relatively identical areas from lobe IX show that propofol treatment increases the thickness of the EGL at P10 compared with the vehicle-treated mice. Data are presented as the mean ± SD (*n* = 5). Scale bar: **(A–C)**: 500 μm, **(D–F)**: 100 μm, and **(G–I)**: 25 μm. **P < 0.01.

HE staining on sagittal vermal sections from the cerebellum was used to analyze the folia structure of the cerebellum at P10 in all the groups (Figures 6A–I). Propofol treatment did not alter the area size at P10 (Vehicle 2.84 ± 0.35 mm^2^; Pro 30 or 60 mg/kg 2.91 ± 0.33 mm^2^ or 2.89 ± 0.14 mm^2^, respectively, *P* > 0.05; *n* = 5; Figure 6J). In identical areas of lobe IX (Figures 6D–F), propofol treatment at both 30 and 60 mg/kg increased the thickness of the EGL compared with the vehicle-treated group (Vehicle 30.66 ± 0.96 μm; Pro 30 or 60 mg/kg 34.06 ± 1.27 μm or 34.73 ± 0.43 μm, respectively, *P* < 0.01; *n* = 5; Figures 6G–I,K).

**Figure 7:**
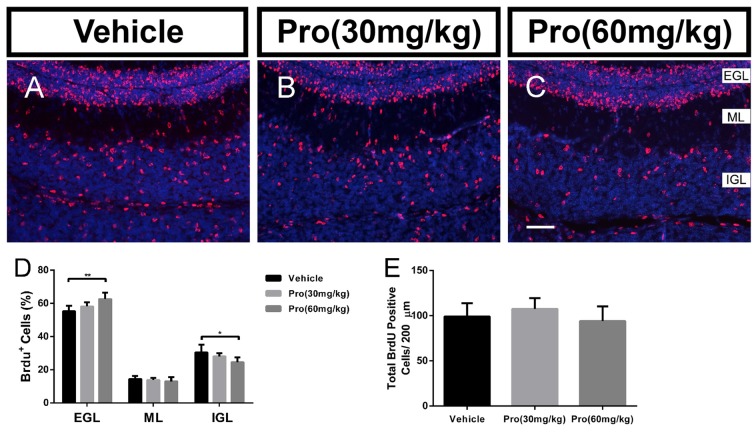
Propofol treatment suppressed the radial migration of the granule neurons from the EGL to IGL. Granule neurons were labeled with BrdU *in vivo* at P8 and the cerebella were harvested at P10 **(A–C)**. Sections were counterstained with DAPI (blue). **(D)** Quantification of the percentage of BrdU-positive cells in EGL, ML, or IGL to the total BrdU-positive cells at P10. **(E)** Quantification of the total number of BrdU-positive cells in EGL, ML and IGL at P10. Data are presented as the mean ± SD (*n* = 5). Scale bar: **(A–C)**: 50 μm. *P < 0.05 and **P < 0.01.

During postnatal development of the cerebellum, the post-mitotic cells in the EGL migrate to their destination in the IGL. The correct positioning of these cells is essential for the final cytoarchitecture and in particular for the three well-defined laminations. It has been suggested that Bergmann glial provided the scaffold for granule neuron migration (Buffo and Rossi, 2013; Xu et al., 2013). BrdU birthdating was used to evaluate the effect of propofol on further granule neuron migration. At P10, the total number of BrdU positive cells was not changed by Propofol treatment (Vehicle 99.05 ± 14.77; Pro 30 or 60 mg/kg 107.40 ± 12.05 or 94.03 ± 16.26, respectively, *P* > 0.05; *n* = 5; Figure 7E) and there was no significant difference in the percent of BrdU-positive cells in the ML in the propofol-treated mice compared to the vehicle-treated mice (Figures 7A–C). While the statistical analysis revealed that propofol treatment (30 or 60 mg/kg) increased the percent of BrdU-positive cells in the EGL by 3% or 7.3%, respectively, compared to the vehicle-treated group (Figure 7D). Meanwhile, the percentage of BrdU-positive cells in the IGL was also decreased significantly by propofol treatment (60 mg/kg) compared with the vehicle-treated mice; in contrast, there were no changes due to propofol administration at the 30 mg/kg dosage (Figure 7D). These results indicate that propofol treatment did not suppress the proliferation, but the migration of granule neurons from the EGL to IGL.

### Propofol Treatment Down-Regulates the Jagged1/Notch Pathway in the Cerebellum

**Figure 8:**
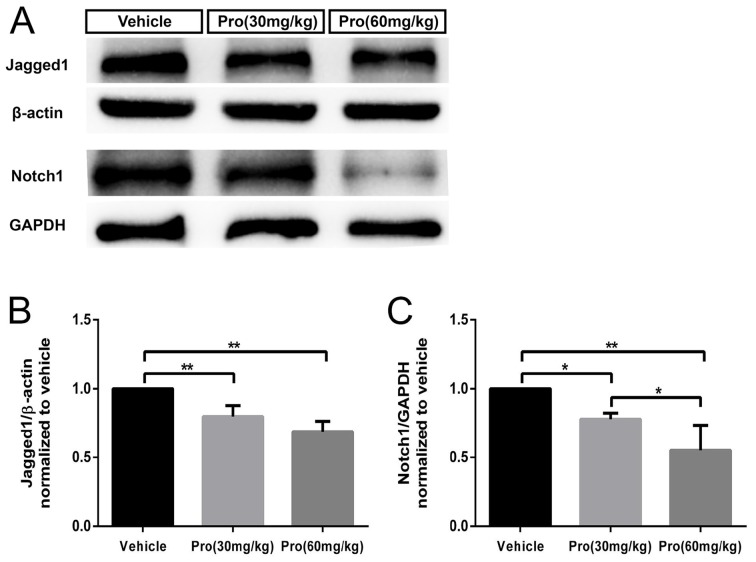
Propofol treatment induced down-regulation of the Jagged1/Notch pathway in the cerebellum at P8. **(A)** Representative western blotting for the Jagged1 and Notch1 proteins from the cerebella in each group. **(B)** Densitometric quantification of Jagged1. Jagged1 protein levels in the Propofol (30 mg/kg)- and Propofol (60 mg/kg)-treated groups were significantly lower than in the vehicle-treated group. **(C)** Densitometric quantification of Notch. Notch1 protein levels in the Propofol (30 mg/kg)- and Propofol (60 mg/kg)-treated groups were significantly lower than in the vehicle-treated group. *P < 0.05 and **P < 0.01.

It has been reported that BLBP protein is a direct target of Notch signaling (Anthony et al., 2005) and active Notch1 signaling is involved in radial fiber formation of Bergmann glia (Xu et al., 2013). The loss of Jagged1, a ligand for Notch signaling, induced a reduction in the number of Bergmann glia cells and affected their morphology (Tanaka and Marunouchi, 2003; Weller et al., 2006). We found that Jagged1 and Notch1 levels were considerably decreased in the cerebella at P8 following exposure to propofol at the 30 mg/kg dose compared to vehicle treatment; a further reduction was detected after treatment with the 60 mg/kg dose (Figure 8A). Quantitative analysis showed that propofol treatment (30 or 60 mg/kg) decreased Jagged1 expression by 20% or 32%, respectively, compared with the vehicle-treated group (Figure 8B); Notch1 protein levels were decreased by 22% and 45%, respectively (Figure 8C). These results suggested that propofol impaired the morphogenesis and phenotypic differentiation of Bergmann glia, potentially via Jagged1/Notch1 signaling.

## Discussion

In this study, we studied the effects of propofol exposure on cerebellar development during early life. Our results demonstrated that a single injection of propofol at a dosage of 60 mg/kg led to reduced Purkinje cell dendritogenesis, retarded granule cells migration, and suppressed radial glia phenotypic differentiation to Bergmann glia cells. The unbalanced transformational process demonstrated by decreased glial fibers in the ML and increased GFAP-positive astrocytes in the white matter may be due to inhibition of Notch signaling. Propofol at a lower dose of 30 mg/kg resulted in a less pronounced interruption in cerebellar development, with no significant influences on Purkinje cell morphogenesis.

Purkinje neurons are GABAergic neurons and considered to grow postnatally. As the sole output neuron in the cerebellar circuit, Purkinje neurons are also the major cell group that integrate motor coordination and learning (Van Der Giessen et al., 2008; Lee R. X. et al., 2015). In rodents, Purkinje neurons exhibit cytoarchitectural changes characterized by highly branched dendritic trees during first 2 weeks after birth (Tanaka, 2009). Our previous studies together with other reports have demonstrated that Purkinje neurons are extremely vulnerable to the neurotoxic effects of EtOH exposure during the early postnatal period (Yang Y. et al., 2014). Emerging evidence has found that propofol administration to neonatal animals caused significant cell loss in the hippocampus (Han et al., 2015; Huang et al., 2016), a typical laminated structure development postnatally. In this investigation, we demonstrated that propofol exposure significantly decreased Purkinje neurons as assessed by calbindin in the cerebellum at P8 in a dose-dependent manner. We further confirmed that propofol administration into neonatal mice significantly reduced the length of cerebellar Purkinje neuron dendrites in a dose-dependent manner. Taken together, these results indicated that propofol treatment impaired dendritic growth in Purkinje neurons.

Bergmann glia, which are normally located in the PCL, extend radial fibers stretching from the cell body towards the pial surface. The radial glial cells originate from the ventricular neuroepithelium (VN), migrate and differentiate to all cerebellar glia, including the Bergmann glia, a specialized subtype of the astrocyte (Buffo and Rossi, 2013). It has been indicated that Bergmann glial cells are arranged around the cell bodies of Purkinje neurons at P8 (Xu et al., 2013). Both BLBP and GFAP are specific markers for Bergmann glia, and immunofluorescence staining showed that the number of Bergmann glial fibers in mice exposed to propofol was significantly decreased and that the radial fibers could not extend to the pial surface. Moreover, GFAP-positive, star-shaped astrocytes were increased in the IGL and deep white matter of the cerebella after propofol treatment at the 60 mg/kg dose. These data implied that propofol might accelerate the transformation of radial glial cells into astrocytic phenotype, with star-shaped bushy processes, rather than Bergmann glia with filiform processes. Indeed, Bergmann glia extend long radial fibers in synchrony with the growth of Purkinje cell dendrites during postnatal development. Thus, Bergmann glial fibers may specifically contribute to Purkinje cell dendrite development (Bellamy, 2006). Recent studies indicated that Bergmann fibers enwrapped the synapses in parallel and climbing fibers interact with Purkinje cells affecting Purkinje cell dendrite arborization (Yamada et al., 2000; Lordkipanidze and Dunaevsky, 2005). Consistent with previous reports, we noticed that suppressed Bergmann glial cell filiform processes and their alignment from propofol exposure led to decreased attachments between Purkinje cell dendritic tips and glial fibers. Hence, it inferred that Bergmann glial fibers loss might contribute to propofol-induced suppressed Purkinje cell dendritogenesis.

The creation of laminated postnatal cerebellar cortex structures is achieved through the directional migration of committed granule neurons along the Bergmann glial radial fibers from the EGL to their destination in the IGL (Sillitoe and Joyner, 2007; Qiu et al., 2010). The intimate structural associations between granule neurons and Bergmann glial fibers are crucial for granule neuron migration. Moreover, several abnormalities in the Bergmann glial fiber radial scaffold structure have been shown to cause granule cell migration alterations (Shetty et al., 1994; Qu and Smith, 2005; Yue et al., 2005; Lin et al., 2009; Nguyen et al., 2013). Propofol induced a thicker EGL and increased number of granule cells remaining in the EGL, which suggested that granule cell migration was retarded. We found that granule cell migration from the EGL to IGL was markedly suppressed by using BrdU birthdating. The lower efficiency of migration could be due to physical impediments to the Bergmann fibers. It is also possible that some granule neurons migrate normally along adequate fibers; hence, only a subpopulation is dramatically retarded in their migration.

Bergmann glia development is regulated by several transcription factors and nuclear receptors (Xu et al., 2013). It has been reported that the Notch signaling pathway was an important factor involved in Bergmann glial differentiation and maturation (Lutolf et al., 2002; Tanaka and Marunouchi, 2003). Additionally, the Notch signaling pathway regulates early events in radial formation through direct cell-cell contacts and is necessary for neuronal-induced radial glia formation (Weinmaster, 1997; Gaiano et al., 2000). An *in vitro* study has confirmed that Notch pathway activation induced cerebellar astroglia to adopt radial glia morphologies (Patten et al., 2003). Many studies have demonstrated that Notch or Jagged ablation in mice promoted a reduced number of Bergmann glia and abnormal Bergmann processes, which led to further deceleration of granule cell migration and impaired Purkinje cell development (Lutolf et al., 2002; Weller et al., 2006; Komine et al., 2007). In this study, we found that both Notch1 and Jagged1 levels were decreased by propofol treatment, even at the low dose, indicating that the Notch signaling pathway was active early in the inhibition of Bergmann glia development induced by propofol exposure.

It has been raised that mechanism of propofol toxicity in immature neurons was GABAergic via the GABA_A_ receptor (Kahraman et al., 2008). GABA_A_ receptor is widely distributing in the cerebellum, including granule cells, Bergmann cells, Purkinje cells, stellate/basket cells and so on (Laurie et al., 1992). What’s more, the GABA_A_ receptors in Bergmann glia express more in the early development period and then decrease in the adulthood (Müller et al., 1994). It highly matches the enhanced activity of Bergmann glia in granule cell migration and synapse formation or remodeling in the early time. Riquelme et al. (2002) found 50% of the Purkinje dendritic spines with the neuronal GABA_A_ receptors were wrapped by Bergmann glia fibers contained GABA_A_ receptors. Ango et al. (2008) further confirmed the dendritic-targeting GABAergic stellate axons are guided to Purkinje dendrites by the Bergmann fibers scaffold and crucial in the physiological control of synaptic integration in postsynaptic neurons. GABA_A_ receptor indeed plays an important role in Bergmann glia function. Therefore, the influence of propofol on Bergmann glia or the whole cerebellum via GABA_A_ receptor deserved more consideration, and the relationship between GABA_A_ receptor and Notch pathway needed to be further explored.

In conclusion, our data indicate that propofol treatment during the early postnatal time significantly impaired Bergmann glial cell development and filiform processes, and led to inhibition of Purkinje cell morphogenesis and granule cells migration afterwards. This study indicates that the cerebellum is sensitive to neurotoxicity induced by high doses of propofol. These findings suggest that propofol used in neonates or young children should be monitored more carefully.

## Author Contributions

RX and DY conducted the experiments, collected and analyzed the data and drafted the manuscript; XL, JH, SJ and XB contributed to acquisition and analysis of data; TY and XF designed the experiments, supervised the project and revised the manuscript.

## Conflict of Interest Statement

The authors declare that the research was conducted in the absence of any commercial or financial relationships that could be construed as a potential conflict of interest.

## Abbreviations

BLBP: Brain lipid binding protein
BrdU: 5-Bromo-2′-deoxyuridine
BSA: Bovine serum albumin
CB: Calbindin
DAPI: 4′,6-diamidino-2-phenylindole
EGL: External granular layer
GFAP: Glial fibrillary acidic protein
HE: Hematoxylin-eosin
IGL: Internal granular layer
IOD: Integrated optical density
i.p.: intraperitoneally
ML: Molecular layer
NC: Nitrocellulose
NeuN: Neuronal nuclei
PBS: Phosphate-buffered saline
PCL: Purkinje cell layer
ROD: Relative optical density
RT: Room temperature.
